# WNT5A-Induced Activation of the Protein Kinase C Substrate MARCKS Is Required for Melanoma Cell Invasion

**DOI:** 10.3390/cancers12020346

**Published:** 2020-02-04

**Authors:** Purusottam Mohapatra, Vikas Yadav, Maren Toftdahl, Tommy Andersson

**Affiliations:** Cell and Experimental Pathology, Department of Translational Medicine, Clinical Research Centre, Skåne University Hospital, Lund University, SE-202 13 Malmö, Sweden; vikas.yadav@med.lu.se (V.Y.); maren.toftdahl@gmail.com (M.T.)

**Keywords:** melanoma, invasion, WNT5A, MARCKS, phosphorylation, MANS peptide

## Abstract

WNT5A is a well-known mediator of melanoma cell invasion and metastasis via its ability to activate protein kinase C (PKC), which is monitored by phosphorylation of the endogenous PKC substrate myristoylated alanine-rich c-kinase substrate (MARCKS). However, a possible direct contribution of MARCKS in WNT5A-mediated melanoma cell invasion has not been investigated. Analyses of melanoma patient databases suggested that similar to *WNT5A* expression, *MARCKS* expression appears to be associated with increased metastasis. A relationship between the two is suggested by the findings that recombinant WNT5A (rWNT5A) induces both increased expression and phosphorylation of MARCKS, whereas WNT5A silencing does the opposite. Moreover, WNT5A-induced invasion of melanoma cells was blocked by siRNA targeting MARCKS, indicating a crucial role of MARCKS expression and/or its phosphorylation. Next, we employed a peptide inhibitor of MARCKS phosphorylation that did not affect MARCKS expression and found that it abolished WNT5A-induced melanoma cell invasion. Similarly, rWNT5A induced the accumulation of phosphorylated MARCKS in membrane protrusions at the leading edge of melanoma cells. Our results demonstrate that WNT5A-induced phosphorylation of MARCKS is not only an indicator of PKC activity but also a crucial regulator of the metastatic behavior of melanoma and therefore an attractive future antimetastatic target in melanoma patients.

## 1. Introduction

Melanoma is an aggressive skin cancer in which rapid metastasis leads to a short overall median survival in patients. Initiation of melanoma has been shown to be related to environmental factors but also to genetic factors [1,2]. Most reasonably, it is in most cases a combination of both environmental and genetic factors that causes melanoma initiation and its progression to a metastatic state [3]. Research suggests that multiple pathways are involved not only in the initiation but also in the fatal metastatic spread of melanoma [1]. Therefore, a detailed understanding of different metastatic-related signaling pathways associated with invasive events is crucial for the development of novel therapeutics that would prevent the dissemination of melanoma cells. In this context, WNT5A signaling has been identified as an important cascade in melanoma progression and metastasis [4,5,6,7,8]. However, characterizing the mechanism of WNT5A signaling in melanoma metastasis has proven to be challenging and, consequently, knowledge of the downstream molecular partners of WNT5A signaling is still incomplete. The identification of downstream targets of WNT5A signaling would allow the development of a combined and more effective treatment strategy in which not only WNT5A signaling is directly targeted, but also essential downstream effector(s) of this signaling pathway.

WNT5A modulates melanoma cell behavior via multiple cell surface receptors/co-receptors, including ROR2 [9] and Frizzleds [10], and downstream molecular targets, such as APT1 [11], IL-6 [12], and PKC-STAT3 [13], which in turn affect various melanoma cell functions, including migration and invasion. One clear example of the essential role of WNT5A signaling in melanoma cell dissemination is the observation that simultaneous inhibition of WNT5A expression (with an anti-IL-6 antibody) and downstream WNT5A signaling (with the WNT5A antagonistic peptide Box5) effectively impairs melanoma cell migration and invasion [12]. An important pathway that has been shown to be crucial for WNT5A-mediated melanoma invasion and metastasis is the protein kinase C (PKC) signaling pathway, as demonstrated in several studies by Weeraratna and coworkers [8,9,14]. One plausible explanation for why PKC signaling is important for WNT5A-induced invasion of melanoma cells comes from the finding that WNT5A/PKC signaling causes epithelial-mesenchymal-transition (EMT)-like changes in melanoma cells [14], a transformation well known to increase tumor cell invasiveness and metastasis [15]. Another alternative downstream target of WNT5A-induced PKC signaling in melanoma cells that so far has only been used to monitor PKC activity in these cells is the phosphorylation of the endogenous PKC substrate myristoylated alanine-rich C-kinase substrate (MARCKS) [16].

MARCKS is a membrane-bound protein that functions in various important cellular processes, such as cytoskeletal remodeling, motility, secretion and exocytosis [17]. For instance, MARCKS has been shown to regulate the actin cytoskeleton and, consequently, the number and length of filopodia [18]. Interestingly, the phosphorylated form of MARCKS has been shown to promote cell motility and membrane protrusions and significantly affect the invasiveness of several cancer cells [17]. However, a possible functional role of the MARCKS protein in WNT5A-induced melanoma cell migration and invasion has not yet been studied.

The aim of the present study was to investigate a potential downstream regulatory role of the MARCKS protein in WNT5A-mediated invasion of melanoma cells. Our results demonstrate that WNT5A-induced activation of MARCKS is a necessary molecular event that is crucial for the metastatic behavior of melanoma cells.

## 2. Results

### 2.1. MARCKS Expression and Prognostic Value in Melanoma Tissue and Cells

To evaluate a possible role of MARCKS in melanoma progression, we first investigated its expression in normal versus melanoma tissue from patients included in the Oncomine database [19]. The Oncomine database contains cancer microarray results, in which transcriptome data can be compared with respective normal tissue data for most cancers and their subtypes. Our Oncomine-based analyses revealed that MARCKS mRNA expression is higher in patient-derived melanoma tissue compared to normal skin tissue samples (Figure S1A,B). To answer the question of whether and how the melanoma expression of MARCKS is related to prognosis, we analyzed the TCGA melanoma (TCGA-SKCM) cases using the “R2: genomics analysis and visualization platform” (http://r2.amc.nl). We have used the “scan modus” cutoff mode of the Kaplan Meier (KM) plot option of the “R2: genomics analysis and visualization platform” which according to the developers of the R2: platform, conducts the sample grouping in a more significant manner compared to other cutoff modes included in the R2: platform. Unfortunately, with this cutoff mode one only obtains a small number of samples with high MARCKS expression (Figure S1C). Therefore, it is very difficult to draw a solid conclusion with regard to how MARCKS expression relates to overall survival of melanoma patients. However, to gain further insight into the cellular mechanisms behind the observation that MARCKS expression could predict the prognosis of melanoma patients, we used the HOPP melanoma cell line database and analyzed the association of MARCKS expression with melanoma cell proliferation and invasion [20]. As shown in Figure S1D, MARCKS expression was significantly related to melanoma cell invasion, as evidenced by the increased expression levels of MARCKS mRNA in the invasive melanoma cell lines in comparison with those in proliferative melanoma cell lines (Figure S1D). Taken together, our in silico analyses suggest a possible role for MARCKS in promoting melanoma metastasis and thereby reducing the survival of melanoma patients.

### 2.2. Correlation between MARCKS and WNT5A Expression

The finding that MARCKS expression relates to the progression of melanoma is comparable to that found for WNT5A expression in relation to melanoma progression. Therefore, we next explored a possible association between MARCKS and WNT5A expression. We first analyzed the correlation between MARCKS and WNT5A in the TCGA database melanoma samples using the www.cbioportal.org data visualization and analysis website. Interestingly, our results revealed a weak positive correlation between MARCKS and WNT5A mRNA expression in melanoma patient tissue (Figure S2A). The above observation led us to investigate how the expression of MARCKS and WNT5A proteins correlated in melanoma cell lines with different WNT5A levels. The results revealed that all four melanoma cell lines (WM852, HTB63, A375 and A2058) used in our study expressed significant levels of MARCKS irrespective of their WNT5A levels (Figure S2B–D). Interestingly, WM852 melanoma cells which had higher WNT5A levels (Figure S2B,C) expressed significantly elevated amounts of MARCKS when compared to the other melanoma cell lines (HTB63, A375 and A2058 cells) (Figure S2B,D). Although we did not find a strong correlation between MARCKS and WNT5A expression, there are other possibilities whereby these two molecules can be related, for example, via a WNT5A-induced phosphorylation of the MARCKS protein [16].

### 2.3. WNT5A Signaling Increases MARCKS Phosphorylation in Melanoma Cells

First, we evaluated the levels of MARCKS phosphorylation at Ser-159/163 and Ser-167/170 sites in different melanoma cell lines via Western blotting approach (Figure S3). Interestingly, we observed that the Ser-159 and Ser-163 phosphorylation levels of MARCKS were significantly higher when compared to the Ser-167 and Ser-170 phosphorylation levels in all tested melanoma cell lines (Figure S3). Our results support previous reports on MARCKS Ser-159 and -163 phosphorylation [21]. Based on these results, we decided to focus on the Ser-159/163 phosphorylation of the MARCKS protein and how it relates to WNT5A signaling and melanoma cell invasion.

**Figure 1:**
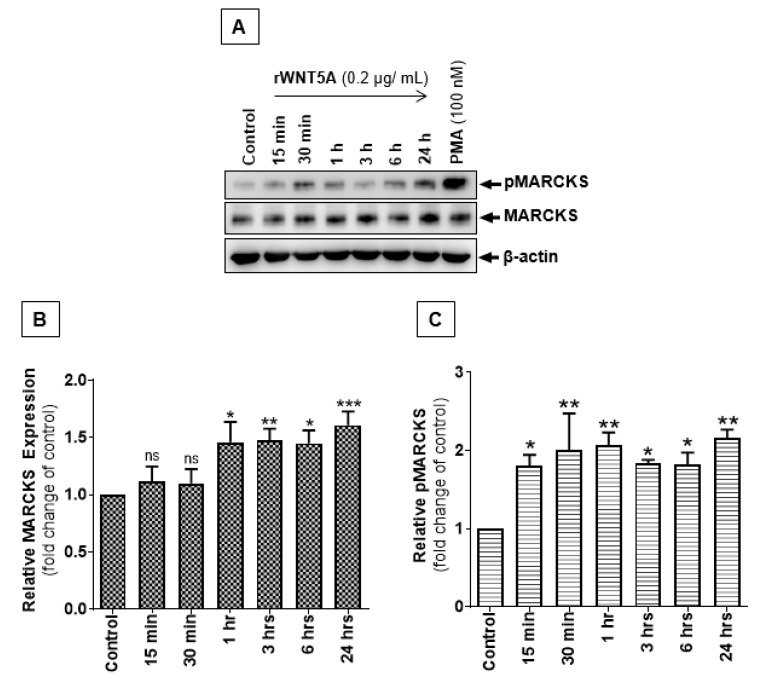
WNT5A signaling increases the expression and phosphorylation of MARCKS in melanoma cells. Western blotting was performed as detailed in the materials and methods section to demonstrate the importance of WNT5A signaling in MARCKS regulation. (**A**) Western blots showing the effect of 0.2 µg/mL rWNT5A treatment in A2058 melanoma cells at the indicated time points on both the expression and phosphorylation of MARCKS. β-Actin was used as a loading control. (**B**,**C**) The graphs represent the densitometry analysis of (**B**) total MARCKS and (**C**) pMARCKS Ser-159/163 levels in A2058 melanoma cells. The results (n = 4) are presented as the means ± S.E.M.; *, *p* < 0.05, **, *p* < 0.001, and ***, *p* < 0.001.

Although it has been previously reported that WNT5A/PKC signaling is directly involved in melanoma cell metastasis via an epithelial-mesenchymal-like transition [8,9,14], the possibility that WNT5A signaling promotes the invasiveness of human melanoma cells via altered expression or phosphorylation of MARCKS has not yet been studied. Here, A2058 (Figure 1A–C) and A375 (Figure S4A–C) melanoma cells were exposed to 0.2 μg/mL recombinant WNT5A (rWNT5A) protein for different time periods (starting at 15 minutes to a maximum of 24 hr) to observe any changes in total expression and Ser-159/163 phosphorylation levels of MARCKS. Phorbol myristate acetate (PMA), a PKC activator, was used as a positive control for MARCKS phosphorylation. Interestingly, the expression of both total MARCKS and phosphorylated MARCKS (Ser-159/163) increased with increasing time periods of rWNT5A treatment in both A2058 (Figure 1A–C) and A375 (Figure S4A–C) cells. The WNT5A-mediated increase in total MARCKS expression was statistically significant in A2058 cells after 1 h (Figure 1B) and in A375 melanoma cells after 3 h (Figure S4B) of treatment with rWNT5A. However, the increase in MARCKS phosphorylation at Ser-159/163 was statistically significant after 15 minutes of rWNT5A treatment in both A2058 (Figure 1C) and A375 melanoma cell lines (Figure S4C). These results clearly suggest that MARCKS phosphorylation at the Ser-159/163 residues is regulated by WNT5A signaling in metastatic melanoma cells. 

### 2.4. The MARCKS Protein Is Important for WNT5A-Mediated Invasion of Melanoma Cells

**Figure 2:**
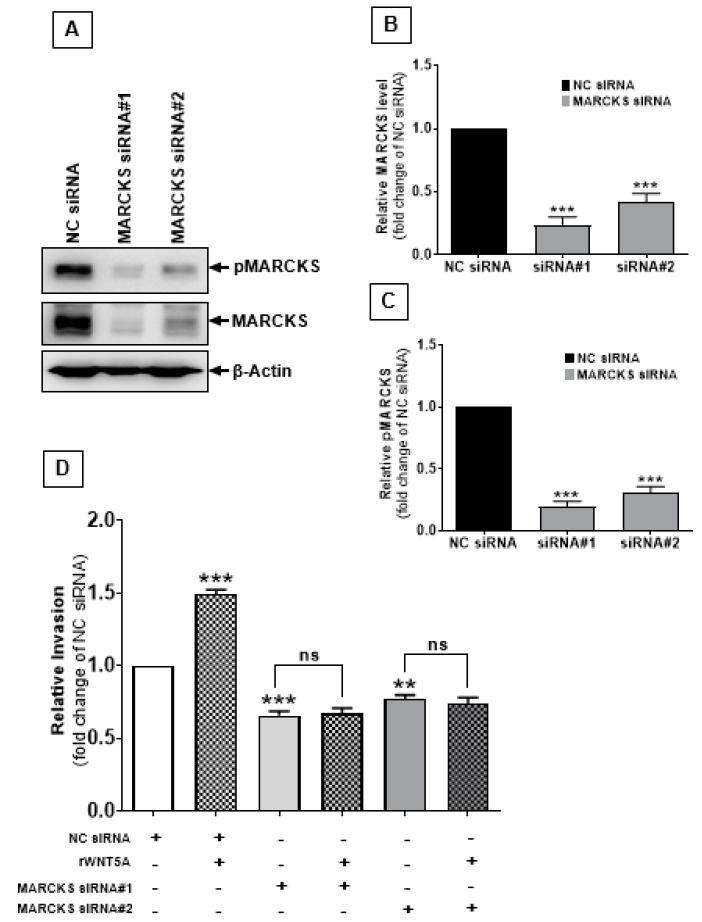
MARCKS is important for WNT5A-mediated melanoma cell invasion. (**A**) Western blot analysis of MARCKS and pMARCKS Ser-159/163 in A2058 melanoma cells transfected with two different MARCKS siRNAs as described in the materials and methods section. β-Actin was used as a loading control. (**B**,**C**) The graphs represent densitometry analyses of (**B**) MARCKS and (**C**) pMARCKS S159/163 levels. The results (n = 4) are presented as the means ± S.E.M.; ***, *p* < 0.001. (**D**) Transwell invasion assays were performed to determine the effect of rWNT5A (0.2 µg/mL) on the invasive capacity of MARCKS-silenced A2058 melanoma cells. The numbers of invaded cells were quantified using the NIH ImageJ software, and the results are presented as relative invasion. The results (n = 3) are presented as the means ± S.E.M.; **, *p* < 0.001, and ***, *p* < 0.001.

Based on the above results, we speculated that WNT5A-mediated melanoma cell invasion could be directly dependent on MARCKS expression and/or its phosphorylation. A2058 melanoma cells expressing very low amounts of WNT5A but with significant expression of the MARCKS protein (Figure S2B–D) were used to test whether the WNT5A-induced melanoma cell invasion was dependent on the presence of the MARCKS protein. MARCKS expression was reduced in A2058 melanoma cells by two different MARCKS siRNAs treatments (Figure 2A–C). Interestingly, stimulation with rWNT5A caused an increase in the numbers of invasive cells, whereas MARCKS silencing led to a 30–40% reduction in A2058 melanoma cell invasion compared to the control siRNA-transfected cells (Figure 2D). Induction of WNT5A signaling via treatment with rWNT5A significantly increased the number of invasive A2058 cells. Interestingly, however, we observed that rWNT5A exposure could not rescue the anti-invasive effect of MARCKS siRNA silencing in A2058 melanoma cells (Figure 2D). Importantly, these results did not discriminate as to whether it was the expression or the phosphorylation status of MARCKS that is crucial for WNT5A-induced melanoma cell invasion.

**Figure 3:**
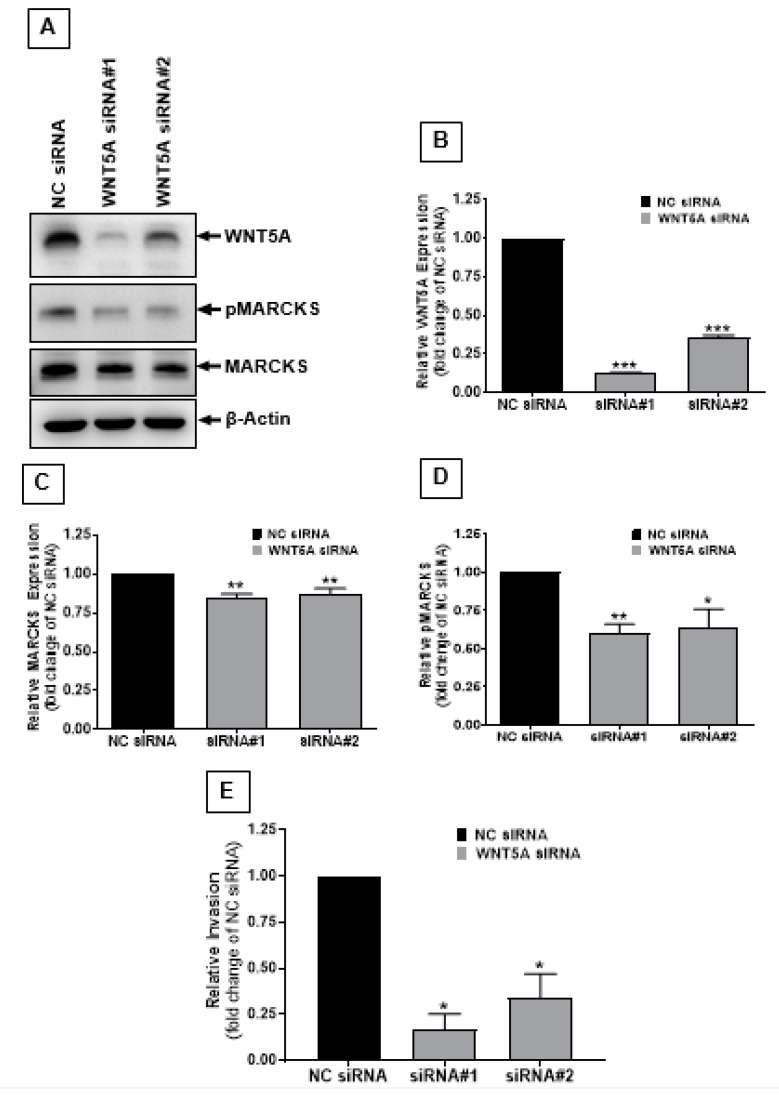
Inhibition of WNT5A signaling simultaneously reduced cell invasion and the expression and phosphorylation of MARCKS in melanoma cells. (**A**) Western blot analyses of MARCKS and pMARCKS Ser-159/163 in HTB63 melanoma cells transfected with two different WNT5A siRNAs as described in the materials and methods section. β-Actin was used as a loading control. (**B**–**D**) The graphs represent the densitometry analysis of (**B**) WNT5A expression, (**C**) MARCKS expression and (**D**) pMARCKS Ser-159/163 levels in WNT5A siRNA-transfected HTB63 melanoma cells. The results (n = 4) are presented as the means ± S.E.M.; *, *p* < 0.05, **, *p* < 0.001, and ***, *p* < 0.001. (**E**) Transwell invasion assays were performed to study the effect of siRNA-mediated inhibition of WNT5A signaling on the invasive capacity of HTB63 melanoma cells. The numbers of invaded cells were counted using the NIH ImageJ software, and the results are presented as the relative invasion compared to control siRNA. The results (n = 5) are presented as the means ± S.E.M.; *, *p* < 0.05.

To test the above results, we decided to take an opposite approach—that is, we reduced WNT5A signaling and studied its effect on MARCKS expression and phosphorylation. At the same time, we checked the effect of WNT5A silencing on melanoma cell invasion. We silenced WNT5A in HTB63 melanoma cells with two different WNT5A siRNAs (Figure 3) and observed that there was only a minor effect on the total MARCKS level (Figure 3A,C). Interestingly, the Ser-159/163 phosphorylation of MARCKS (Figure 3A,D) was significantly decreased after WNT5A knockdown in HTB63 melanoma cells. As expected, our invasion assay revealed that WNT5A silencing decreased the invasive capacity of HTB63 melanoma cells (Figure 3E).

### 2.5. Direct Inhibition of MARCKS Phosphorylation Blocks WNT5A-Mediated Melanoma Cell Invasion

**Figure 4:**
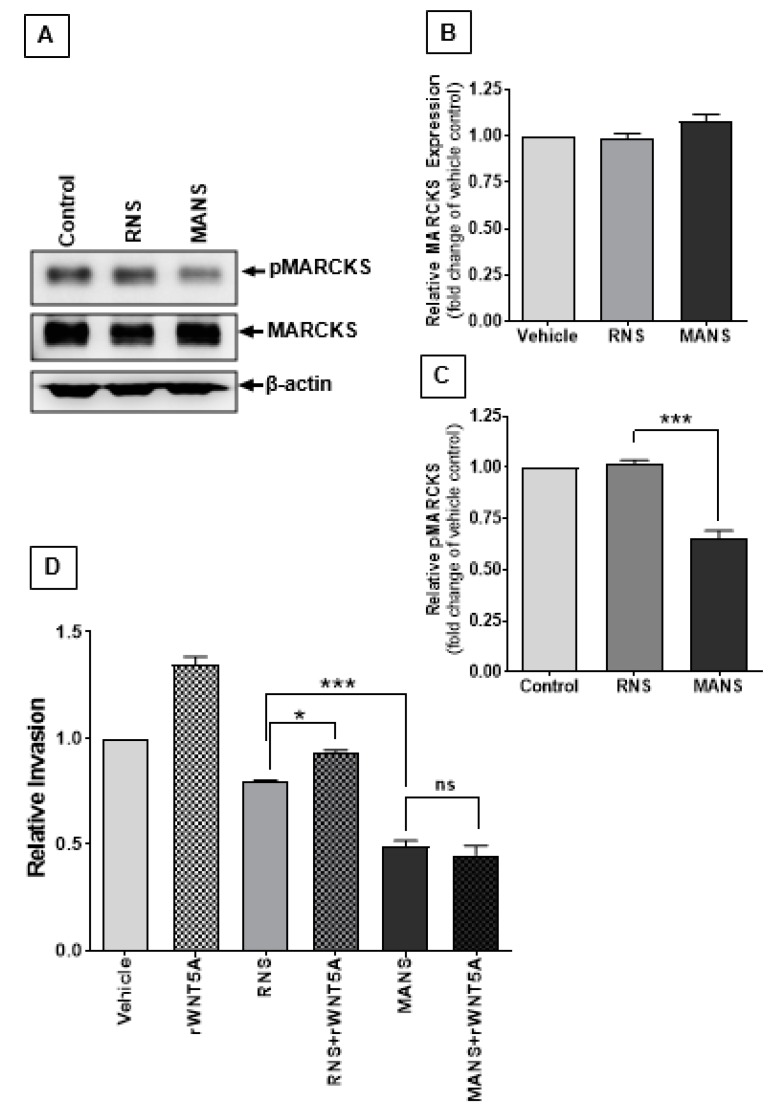
MARCKS phosphorylation-specific peptide MANS blocks WNT5A-induced melanoma cell invasion. Western blotting and transwell invasion assays were performed to evaluate the specific role of MARCKS phosphorylation in WNT5A-promoted melanoma cell invasion. (**A**) Western blots showing the expression of total MARCKS and levels of pMARCKS Ser-159/163 in 100 µM RNS- or MANS peptide-treated A2058 melanoma cells. Representative blots of five separate experiments are shown here. (**B,C**) The graph represents the densitometry analysis of (**B**) MARCKS and (**C**) pMARCKS Ser-159/163 levels normalized against total MARCKS levels. The results (n = 5) are presented as the means ± S.E.M.; ***, *p* < 0.001. (**D**) Effect of the direct inhibition of pMARCKS on WNT5A-induced melanoma cell invasion was evaluated by transwell cell invasion assay as mentioned in the materials and methods section. (**D**) Graph showing the relative melanoma cell invasion of rWNT5A unstimulated/stimulated and RNS peptide- or MANS peptide-treated A2058 melanoma cells. The results (n = 4) are presented as the means ± S.E.M.; *, *p* < 0.05, and ***, *p* < 0.001.

To evaluate whether it is the ability of WNT5A to increase the expression of MARCKS or whether it is its ability to elevate the phosphorylation level of MARCKS that is crucial for melanoma cell invasion, we took a direct approach to inhibit MARCKS phosphorylation with a cell-permeable peptide identical to the MARCKS N-terminus sequence (the MANS peptide), a peptide that does not affect MARCKS expression. We tested the effect of MANS and a control peptide on WNT5A-induced A2058 melanoma cell invasion. Previously, the MANS peptide has been shown to inhibit basal lung cancer cell migration and invasion by specifically inhibiting MARCKS Ser-159/163 phosphorylation [22]. We first verified the expression of total MARCKS and phosphorylated MARCKS (Ser-159/163) after treatment with 100 μM RNS (control peptide) or 100 μM MANS (MARCKS phosphorylation inhibitory peptide) (Figure 4A–C). The phosphorylated MARCKS Ser-159/163 levels were reduced by approximately 35% in MANS peptide-treated A2058 melanoma cells compared to cells treated with the RNS control peptide (Figure 4A,C). However, treatment with either of these peptides did not change the expression levels of MARCKS in A2058 cells (Figure 4A,B). Our observations suggest that the MANS peptide can effectively and specifically reduce the phosphorylation of MARCKS in melanoma cells. Interestingly, we observed that the MANS peptide reduced the invasion of A2058 melanoma cells by 50% compared to that of vehicle-treated cells (Figure 4D). Most importantly, the presence of the MARCKS phosphorylation inhibitory peptide MANS abolished WNT5A-induced A2058 melanoma cell invasion (Figure 4D). The RNS control peptide had a small effect on A2058 cell invasion, which was reversed by rWNT5A treatment (Figure 4D). These observations strongly suggest that WNT5A specifically phosphorylates MARCKS to promote melanoma cell invasion. 

### 2.6. WNT5A Increases Phosphorylated MARCKS Levels at the Cell edge and the Leading Front, Including Cell Protrusions of Melanoma Cells

**Figure 5:**
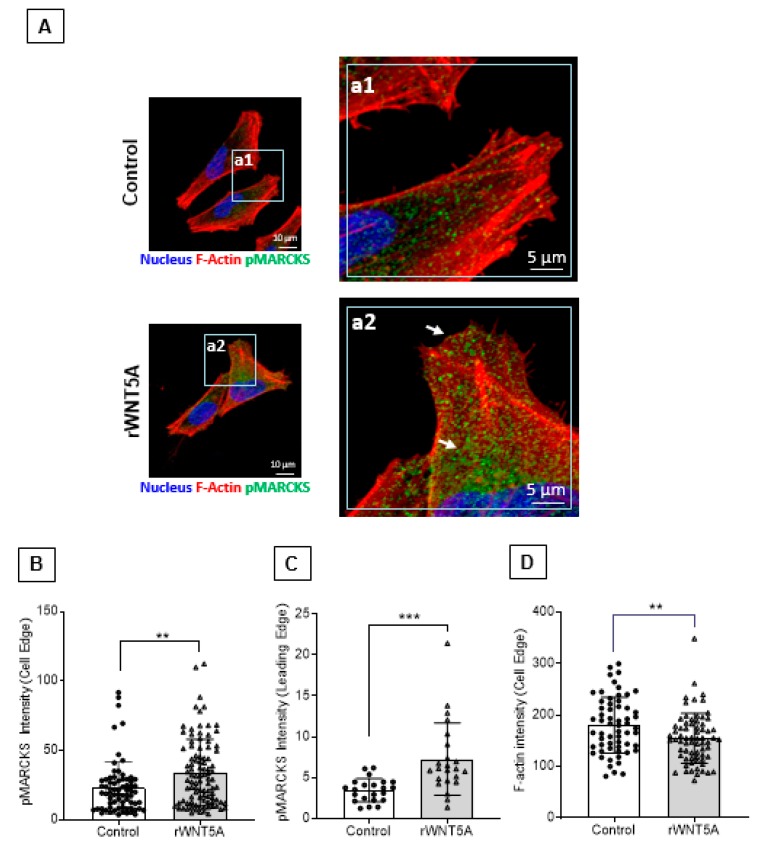
WNT5A signaling promotes the localization of phosphorylated MARCKS to the edge of melanoma cells. (**A**) A2058 melanoma cells were treated with 0.2 µg/mL rWNT5A, and immunofluorescence staining experiments were performed to study the localization of pMARCKS Ser-159/163 as described in the materials and methods section. (**A**) The images on the left show the pMARCKS Ser-159/163 localization in untreated and rWNT5A-treated A2058 melanoma cells. The images to the right identified as a1 and a2 are the magnified region of the marked areas/regions of the left (main) images. Representative images of three independent experiments are shown here. The graphs show the (**B**) integrated fluorescence intensity of pMARCKS Ser-159/163 at the cell edge (including the protrusions) in control *vs* WNT5A-treated A2058 melanoma cells quantified using CellProfiler software. (**C**) Integrated fluorescence intensity of pMARCKS Ser-159/163 at the leading edge of control *vs* WNT5A-treated A2058 melanoma cells was quantified using the Zeiss LSM-700 microscope image analysis software (**D**) F-actin intensity at the cell edge of control *vs* WNT5A-treated A2058 melanoma cells was quantified using CellProfiler software. Two-tailed unpaired Student’s t test was performed, and the above results are presented as the means ± S.E.M., **, *p* < 0.05, ***, *p* < 0.001.

It is well known that lamellipodia-like structures at the cell leading edge are essential for the ability of cells to migrate and invade. Based on a previous finding that the MARCKS protein has been implicated in the formation of such structures [23] and our present results, we speculated that WNT5A signaling could promote melanoma cell migration and invasion via translocation of phosphorylated MARCKS to the leading edge of melanoma cells. We investigated A2058 melanoma cells exposed to rWNT5A for any changes in the subcellular localization of MARCKS by confocal microscopy (Figure 5). Interestingly, our quantitative results further revealed that the fluorescence intensity of phosphorylated MARCKS was significantly higher at the cell periphery (Figure 5A,B) and at the leading edge (Figure 5A,C) of A2058 melanoma cells exposed to rWNT5A compared to the vehicle (control)-treated cells. Since MARCKS phosphorylation hinders its actin cross-linking ability, we wanted to study the consequence(s) of WNT5A-mediated MARCKS phosphorylation for the F-actin network of melanoma cells. We quantified the F-actin content at the cell leading edge with high phospho-MARCKS and noticed a significantly decreased fluorescence intensity of F-actin (Figure 5A,D). Overall, these findings add further support for an essential role of phosphorylated MARCKS protein in WNT5A-mediated melanoma cell migration and invasion and indicate that WNT5A-induced translocation of MARCKS to the leading edge of a melanoma cell is an essential step for how WNT5A participates in melanoma cell migration and invasion.

### 2.7. RhoA-ROCK Signaling Also Contributes to WNT5A-Induced Phosphorylation of MARCKS to Increase Invasiveness of Melanoma Cells

**Figure 6:**
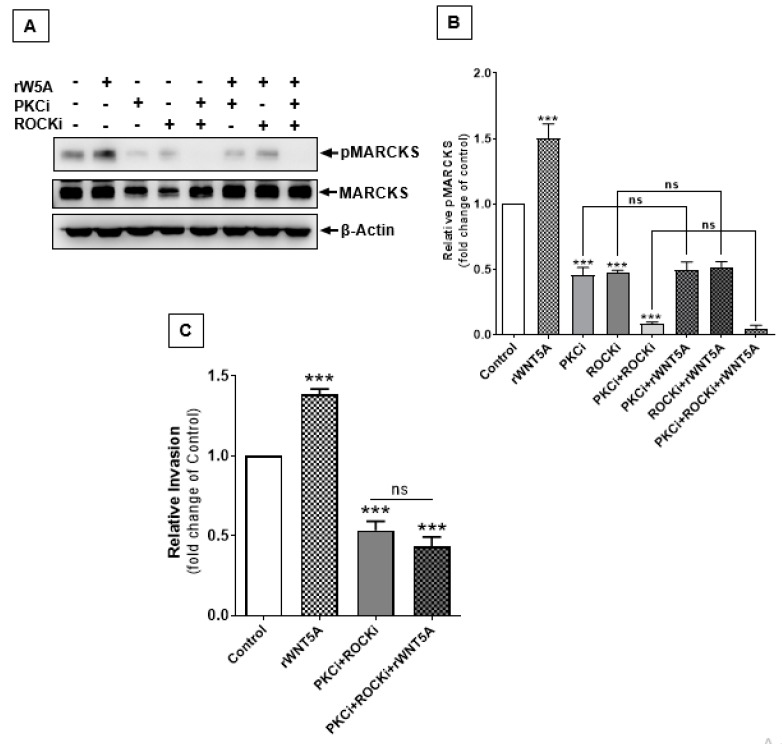
WNT5A-induced RhoA-ROCK signaling also contributes to MARCKS phosphorylation and melanoma cell invasion. Western blotting and transwell invasion assays were performed as described in the materials and methods section to investigate the intermediate signaling regulator of WNT5A-mediated phosphorylation of MARCKS in melanoma cell lines. (**A**) Western blot analysis of A2058 melanoma cells showing the rWNT5A-induced phosphorylation of MARCKS Ser-159/163 in untreated cells and A2058 melanoma cells treated with individual or combination of a PKC inhibitor (Gö6983) and/or ROCK inhibitor (Y-27632). β-Actin was used as a loading control. (**B**) The graph represents the densitometry analysis of phosphorylated-MARCKS normalized against total MARCKS using Bio-Rad ImagePro 6.0 software. The results (n = 5) are presented as the means ± S.E.M.; ***, *p* < 0.001. (**C**) Transwell invasion assays were performed to evaluate the effect of rWNT5A on the invasion capacity of PKC inhibitor- and ROCK inhibitor-treated A2058 melanoma cells. The number of invaded cells was counted using the NIH ImageJ software, and the results are presented as relative invasion. The results (n = 4) are presented as the means ± S.E.M.; ***, *p* < 0.001.

Although MARCKS phosphorylation has previously been used as an indication of WNT5A-mediated PKC activation in melanoma [16], a recent report by Tanabe et al. indicated that RhoA-ROCK signaling can also contribute to MARCKS phosphorylation in human neuroblastoma cells [24]. This prompted us to directly test whether RhoA-ROCK signaling is also involved in WNT5A-mediated phosphorylation of MARCKS in melanoma cells. Our results showed that both a PKC inhibitor and a ROCK inhibitor inhibited approximately 50% of WNT5A-induced MARCKS phosphorylation in A2058 (Figure 6A,B) and A375 (Figure S5A,B) melanoma cells compared to the vehicle-stimulated controls. Interestingly, the combined inhibition of PKC and ROCK signaling caused more than 90% inhibition of MARCKS phosphorylation in both A2058 (Figure 6A,B) and A375 (Figure S5A,B) melanoma cell lines, and this inhibition could not be rescued by cotreatment with rWNT5A. These results indicate that both PKC and RhoA-ROCK are important signals for WNT5A-mediated phosphorylation of MARCKS. Next, the invasion assay results revealed that a combination of a PKC and a ROCK inhibitor blocked the basal invasion of A2058 melanoma cells by at least 50%. More importantly, we observed that rWNT5A treatment had no significant effect on the invasion of A2058 melanoma cells in the simultaneous presence of a PKC and a ROCK inhibitor (Figure 6C). These observations demonstrate a dual signaling phenomenon whereby both the WNT5A-PKC and WNT5A/RhoA-ROCK signaling pathway contribute to MARCKS phosphorylation, thereby dictating its essential role in melanoma cell invasion.

## 3. Discussion

Melanoma is well known for its pronounced metastatic behavior, making this cancer type one of the most aggressive. Several different regulators, including WNT5A and their complex downstream signaling pathways, have been identified to control various aspects of melanoma metastasis. In this study, we identified MARCKS phosphorylation as a novel WNT5A downstream signal crucial for melanoma cell invasion, a key event in the metastatic process.

Although WNT5A/PKC signaling has been previously reported to promote melanoma cell migration and invasion resulting in increased metastasis [14,25], a functional role of the PKC substrate MARCKS has not been studied. Rather, the WNT5A/PKC signal has been shown in melanoma cells to result in an EMT process that was suggested to explain how this signaling pathway caused elevated invasion leading to increased metastasis [14]. Previously, the phosphorylation status of the MARCKS protein has only been used as an indicator of WNT5A-mediated PKC activation in melanoma cells [16]. In the present study, we used online melanoma database analyses to demonstrate that MARCKS expression was increased in melanoma tissue and that it was directly associated with melanoma patient survival and melanoma cell invasiveness. Despite the fact that these databases are based on mRNA analyses and do not necessarily reflect the actual protein levels, these observations suggest that MARCKS expression could be associated with melanoma progression. Interestingly, the database findings on MARCKS are very similar to those of the WNT5A ligand, which is well known for its ability to promote melanoma invasion and metastasis [8] and has been shown to activate PKC [13,14].

By investigating a possible role of the MARCKS protein in melanoma progression and its relation to WNT5A, we found that siRNA silencing of MARCKS resulted in markedly reduced expression of not only total MARCKS but also of phosphorylated MARCKS, in parallel with a reduced invasive capacity of melanoma cells. Most interestingly, the ability of WNT5A to stimulate the invasiveness of MARCKS siRNA-silenced melanoma cells was completely abolished, suggesting an essential WNT5A-downstream signaling role of MARCKS in its ability to induce melanoma cell invasion. This notion was further supported by our findings that siRNA silencing of WNT5A reduced not only the total expression of MARCKS but also the level of phosphorylated MARCKS. Similarly, reconstitution of WNT5A signaling by rWNT5A in cells with very low expression of WNT5A increased not only the total expression of MARCKS but also its phosphorylation level. Although the effector domain of MARCKS, also called the phosphorylation site domain, has, once it is phosphorylated, been reported to mediate the association of the MARCKS protein with other cellular components leading to altered cellular functions [24,26,27], siRNA silencing of MARCKS does not determine if it is the total expression of MARCKS or its phosphorylation that is essential for WNT5A-induced melanoma cell invasion.

Using the MANS peptide, a cell permeable inhibitor of MARCKS phosphorylation [22,28], we were able to specifically abolish MARCKS phosphorylation without altering its expression. This approach allowed us to completely abolish WNT5A-induced melanoma cell invasion, thus demonstrating for the first time a crucial role of MARCKS phosphorylation in WNT5A-driven melanoma cell invasion. Previously, it has been documented that the MANS peptide could effectively block endogenous fibroblast migration by blocking MARCKS phosphorylation [28]. Interestingly, in another report, Chen and coworkers demonstrated that the MANS peptide could reduce the basal level of lung cancer cell invasion [22]. However, these studies focused on the importance of MARCKS for basal invasiveness of cells and not on the possibility that an external ligand could overcome the inhibition of basal cancer cell migration or invasion.

Since MARCKS is an actin-binding protein and phosphorylation of MARCKS could affect actin dynamics and thereby modulate melanoma cell motility and/or invasion, we further studied the molecular and subcellular events during WNT5A-mediated MARCKS phosphorylation in melanoma cells. Our observation of a crucial role of WNT5A-induced MARCKS phosphorylation in regulation of melanoma cell migration and invasion was further substantiated by the findings that MARCKS localized at the cell leading edge and lamellipodia and filopodia-like protrusions, indicating that WNT5A, by inducing MARCKS phosphorylation at the cellular edge, could modulate the cytoskeleton to influence melanoma cell invasion. It has been recently reported that PKC-induced MARCKS phosphorylation impairs cell polarization by destabilizing and redistributing the F-actin network [29]. Although there are no reports showing that WNT5A-induced MARCKS phosphorylation could affect F-actin rearrangement in cancer cells, an interesting study by Iioka et al. demonstrated that noncanonical WNT11 signaling via MARCKS regulates cortical actin dynamics and the formation of lamellipodia- and filopodia-like protrusions [30]. Our observation of a decreased F-actin content at the cell edge suggests that WNT5A signaling by phosphorylating MARCKS interferes with the MARCKS F-actin binding ability and destabilizes the F-actin network at the cell edge to favor melanoma cell invasion.

Interestingly, we demonstrated that inhibition of PKC and ROCK signaling blocked WNT5A-mediated MARCKS phosphorylation and melanoma cell invasion, which indicated that both PKC and RhoA-ROCK, as intermediate signaling pathways, are crucial during WNT5A-regulated MARCKS function. Interestingly, both PKC and RhoA-ROCK signaling are known to lead to MARCKS phosphorylation [17,22,24,29]. Furthermore, PKC and ROCK signaling has been documented as an important event in actin cytoskeletal remodeling, which is essential for cellular rearrangements during convergence, extensions movements [31] and human adipose stem cell regeneration [32]. The present finding that a signaling ligand mediates MARCKS phosphorylation via a simultaneous activation of PKC and RhoA-ROCK pathways has not been previously described in any cancer model. Since MARCKS phosphorylation is important for the control of actin dynamics, our novel observations of PKC and RhoA-ROCK signaling being involved in melanoma cell invasion mediated by WNT5A-induced MARCKS phosphorylation, suggest the importance of these signaling pathways in melanoma. Our study shed light on the previously unknown molecular events associated with WNT5A signaling that regulate MARCKS phosphorylation, which is crucial for melanoma metastasis.

## 4. Materials and Methods

### 4.1. Cell Culture and Reagents 

The WM852 melanoma cell line was procured from the Coriell Institute (Camden NJ, USA) and maintained in RPMI-1640 medium. The HTB63, A375 and A2058 melanoma cell lines were purchased from ATCC (Old Town Manassas, VA, USA). HTB63 melanoma cells were cultured in McCoy’s 5A medium, and both A375 and A2058 melanoma cell lines were cultured in DMEM. The culture medium for all the cell lines was supplemented with 10% FBS, antibiotics and L-glutamine. The supplier confirmed the genetic authentications of all the cell lines, and no cell line was used for more than four years. All cell lines were routinely screened for the absence of mycoplasma infection. The PKC-inhibitor (Gö6983) and ROCK-inhibitor (Y-27632) were procured from Selleckchem (Munich, Germany). The predesigned siRNAs used in this study were obtained from Thermo Fischer Scientific (Waltham, MA, USA).

### 4.2. Western Blotting

Briefly, treated and untreated cells from separate experiments were lysed, and the protein concentration was quantified using the Bradford’s method (Sigma-Aldrich, Stockholm, Sweden). SDS gel electrophoresis was performed, and the protein bands were transferred to a PVDF membrane for Western blotting. Western blotting was conducted using primary mouse anti-MARCKS (Santa Cruz Biotechnology, Dallas, TX, USA; dilution 1:1000), rabbit anti-phospho-Ser-159/163 MARCKS (Cell Signaling Technology, Danvers, MA, USA; dilution 1:1000), rabbit anti-phospho-Ser-167/170 MARCKS (Cell Signaling Technology, dilution 1:500), goat anti-WNT5A (R&D Systems, Minneapolis, MN, USA; dilution 1:100), and anti-β-actin (Sigma-Aldrich, dilution 1:30,000) antibodies and secondary HRP-conjugated rabbit anti-goat, goat anti-mouse or goat anti-rabbit antibodies (Dako, Santa Clara, CA, USA; dilution 1:10,000). Western blot images were obtained using a ChemiDoc^TM^ imaging system (Bio-Rad, Hercules, CA, USA). The densitometry quantifications of relative protein expression were conducted using Image Lab software (version 6.0, Bio-Rad).

### 4.3. MANS and RNS Peptide Synthesis and Treatment

The MANS and RNS peptides were custom synthesized by CASLO ApS (Lyngby, Denmark). The peptides were obtained as lyophilized powder. The MANS peptide is a specific MARCKS phosphorylation inhibitory peptide that consists of a sequence matching the first 24 amino acids of the MARCKS myristoylated N-terminal region [22]. The sequence of the MANS peptide was MA-GAQFSKTAAKGEAAAERPGEAAVA (where MA is the N-terminal myristate chain), and the RNS control peptide sequence was MA-GTAPAAEGAGAEVKRASAEAKQAF, meaning that it had the same amino acid content as the MANS peptide but randomly rearranged. The peptides were dissolved in 1× sterile PBS. A2058 melanoma cells were pretreated for 24 h with 100 µM RNS or MANS peptide before the different experiments were performed as described in the results section and in the figure legends.

### 4.4. Transwell Cell Invasion Assay

The transwell cell invasion method was performed to quantify the number of invaded cells after different treatment conditions. Briefly, 50,000 melanoma cells/inserts were used to compare cell invasive capacities after different treatment/transfection conditions. After treatment, the invaded melanoma cells were fixed in 70% ethanol and stained with crystal violet. The noninvaded cells were removed from the insert membrane using wet cotton swabs followed by washing. Images of the invaded cells were captured using an inverted microscope (Nikon, Tokyo, Japan), and the cell numbers were counted using cell counter plugins of the NIH ImageJ software. 

### 4.5. Transient Gene Silencing Using siRNA

The different melanoma cells were transfected with either MARCKS or WNT5A siRNAs using Lipofectamine 2000 transfection reagent (Invitrogen, Carlsbad, CA, USA) in accordance with the manufacturer’s instructions. All predesigned siRNA oligonucleotides were purchased from Thermo Fischer Scientific (Waltham, MA, USA). The siRNAs were provided as lyophilized powder and resuspended in nuclease-free water (supplied by the manufacturer) prior to transfection. Approximately 400,000 cells/well of either A2058 or HTB63 melanoma cells were transfected with 100 nM negative control (NC) siRNA (#4390843), anti-MARCKS siRNA#1 (s8636), anti-MARCKS siRNA#2 (243176), anti-WNT5A siRNA#1 (s14871), or anti-WNT5A siRNA#2 (s14872) siRNA oligonucleotides.

### 4.6. Immunofluorescence Staining, Confocal Imaging and Image Analysis

Approximately 5000 cells/coverslip of adherent A2058 melanoma cells were treated with 0.2 μg/mL rWNT5A for 1 hr. For phosphorylated MARCKS staining, the cells on the coverslips were fixed with 4% paraformaldehyde for 10 minutes at room temperature, washed with cold 1xPBS, and then blocked in 2% BSA and 0.1% Triton X-100 in 1× PBS for 1 h. After blocking, the cells were incubated in the presence of an anti-phospho-MARCKS (Ser-159/163) antibody (dilution 1:100) overnight at 4 °C. After the primary antibody incubation, the cells were washed three times with cold 1× PBS and incubated with a secondary anti-rabbit antibody conjugated to Alexa Fluor^®^ 488 (Invitrogen, dilution 1:250) and phalloidin-TRITC (Sigma-Aldrich, dilution 1:400) for 1 hr at room temperature in the dark. Cells were washed three times with cold 1× PBS, and their nuclei were counterstained with DAPI. The coverslips containing stained cells were mounted with a fluorescent mounting solution (Dako) on glass slides and kept overnight at 4 °C for curing. The fluorescence images were captured under a 63× oil objective using a confocal microscope (LSM 700, Carl Zeiss, Oberkochen, Germany). Image analyses were performed using the microscope software and CellProfiler image analysis software [33]. The cells clearly showing a leading cell edge were selected from independent experiments to quantify the phospho-MARCKS (Ser-159/163) fluorescence intensity at the leading edge by LSM700 microscope image acquisition and analysis software (Carl Zeiss). Regions of interest (ROIs) at the cell leading edge were marked manually, and the fluorescence intensity values obtained in those regions were used for quantification. The CellProfiler pipelines for fluorescence intensity measurement at the cell edge were set to quantify the fluorescence intensity at the edge of the cell membrane and cell protrusions (integrated fluorescence intensity at the edge) for the localization of phosphorylated MARCKS in rWNT5A untreated and treated cells.

### 4.7. Statistical Analyses

Statistical analyses were performed using GraphPad Prism 8.0 software. All multiple group analyses were verified for significance using ANOVA complemented with Dunnett’s test (when comparing with control) or Bonferroni’s multiple comparison test (when comparing all the groups). Significant differences between two groups were assessed using unpaired two-tailed Students’ *t*-tests. The results are presented as the mean ± S.E.M. Differences were considered significant when *p* < 0.05.

## 5. Conclusions

Our study demonstrates multiple novel findings outlining an essential role of MARCKS phosphorylation in WNT5A-induced melanoma cell invasion. The basis for this conclusion comes from our findings that MARCKS expression might be associated with melanoma progression and patient survival, WNT5A signaling triggers increased expression and phosphorylation of MARCKS, WNT5A increased the localization of phosphorylated MARCKS at the cell leading edge and in its protrusions and, most importantly, blocking MARCKS phosphorylation with the MANS peptide without affecting its expression abolished WNT5A-mediated melanoma cell invasion. These findings provide the basis for a new antimetastatic treatment strategy that involves the simultaneous targeting of WNT5A and MARCKS signaling in malignant melanoma patients. 

## Supplementary Materials

The following are available online at https://www.mdpi.com/2072-6694/12/2/346/s1, Figure S1: MARCKS is positively associated with melanoma progression and poor overall survival of melanoma patients, Figure S2: Metastatic melanoma cells express significant levels of MARCKS and WNT5A, Figure S3: Phosphorylation levels of MARCKS at Ser-159 and Ser-163 are predominant in metastatic melanoma cells, Figure S4: WNT5A signaling increases the expression and phosphorylation of MARCKS in A375 melanoma cells, Figure S5: PKC and RhoA-ROCK signaling are also important for WNT5A-mediated phosphorylation of MARCKS in A375 melanoma cells, Additional Materials Related to Western Blotting Experiments: Whole blots for Western blotting experiments in the main figures are added to this section.
