# Supplementary material for: WNT5A-Induced Activation of the Protein Kinase C Substrate MARCKS Is Required for Melanoma Cell Invasion
Source: Cancers (Basel). 2020 Feb 4;12(2):346. doi: 10.3390/cancers12020346 (PMC7072258; doi:10.3390/cancers12020346)

WNT5A-induced activation of the protein kinase C substrate MARCKS is required for melanoma cell invasion

Purusottam Mohapatra, Vikas Yadav, Maren Toftdahl, Tommy Andersson

Supplementary Figure S1

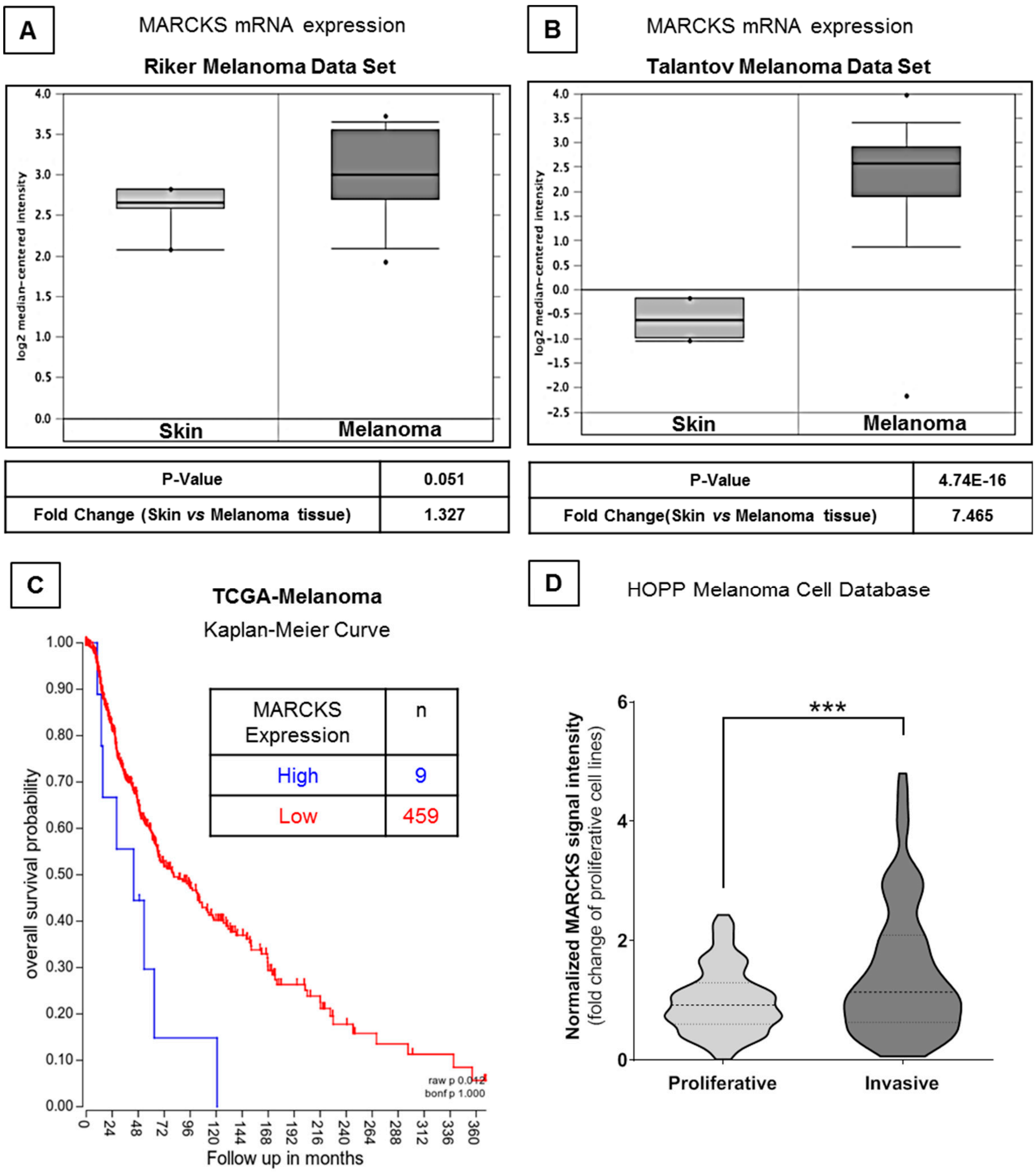

Figure S1: MARCKS is positively associated with melanoma progression and poor overall survival of melanoma patients. The Oncomine and TCGA melanoma online databases were used to analyze the association of MARCKS with melanoma progression and patient survival. Analyses of the **(A)** Riker melanoma data set and **(B)** Talantov melanoma data set from the Oncomine database show the difference in MARCKS mRNA expression between patient-derived normal skin and melanoma tissue. The tables below the graphs present the p-value and fold change of MARCKS mRNA expression. **(C)** TCGA melanoma cases were used to evaluate the correlation between MARCKS expression with overall

melanoma patient survival. The Kaplan-Meier curve indicates the correlation of MARCKS expression (high or low) with melanoma patient overall survival (in months). **(D)** The Heuristic Online Phenotype Prediction (HOPP) melanoma cell line database was used to analyze and compare MARCKS expression in proliferative and invasive melanoma cells. **(D)** The violin plot shows the comparison of the MARCKS mRNA expression in proliferative versus invasive melanoma cell lines using the HOPP melanoma database. The results are presented as the means ± S.E.M.; ***, $p<0.001$.

Supplementary Figure S2

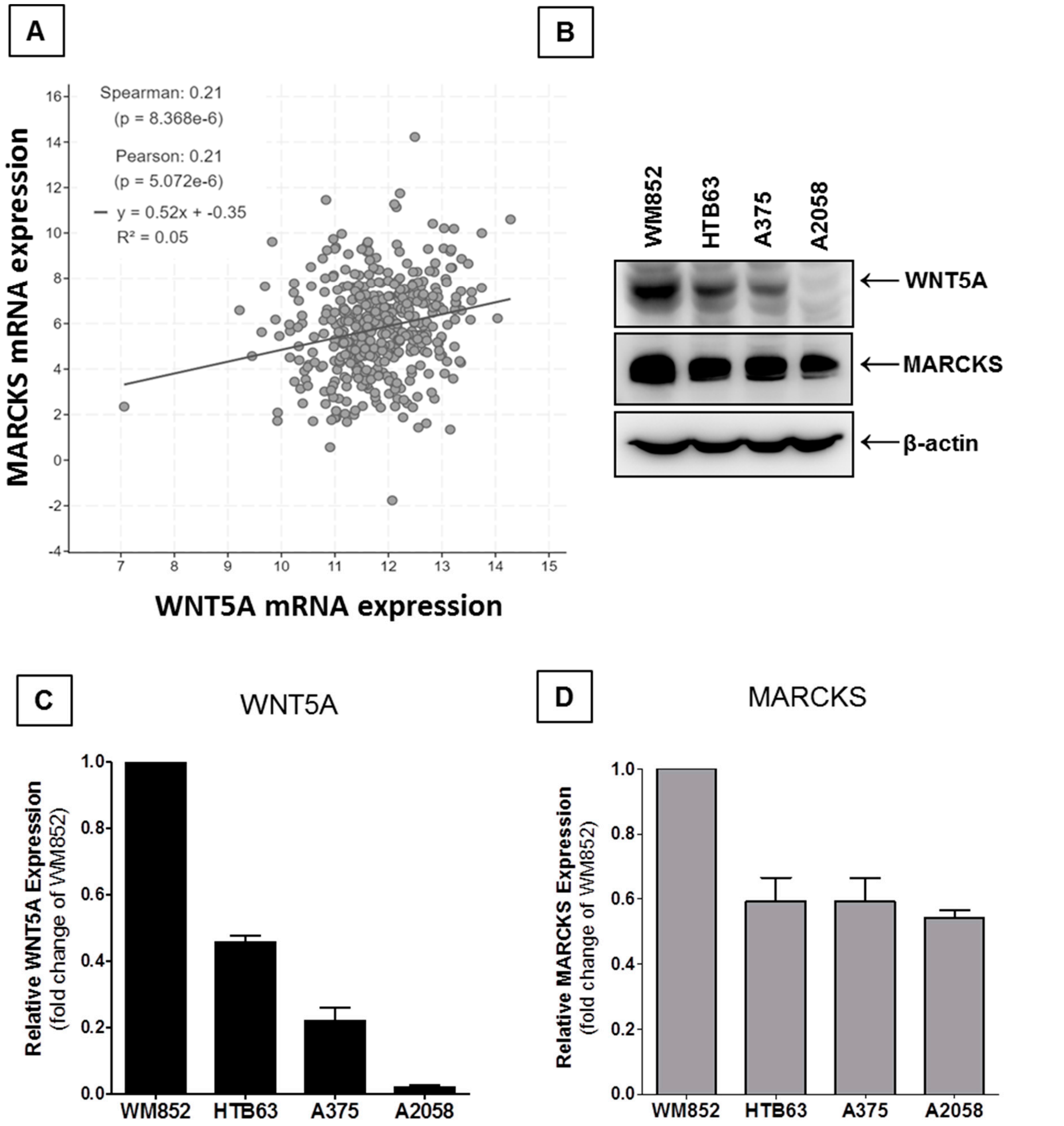

Figure S2: Metastatic melanoma cells express significant levels of MARCKS and WNT5A. (A) The TCGA melanoma database was used to analyze the correlation between *WNT5A* and *MARCKS mRNA* expression. **(B-D)** Western blotting was performed as mentioned in the main materials and method section to evaluate the correlation between WNT5A and MARCKS protein expression in different melanoma cells. **(B)** Representative western blots from three independent experiments show the levels of WNT5A and MARCKS expression in WM852, HTB63, A375 and A2058 melanoma cells. β-Actin was used as a loading control for these experiments. Graphs represent the densitometry analyses of **(C)** WNT5A and **(D)** MARCKS protein expression normalized against that of β-actin.

Supplementary Figure S3

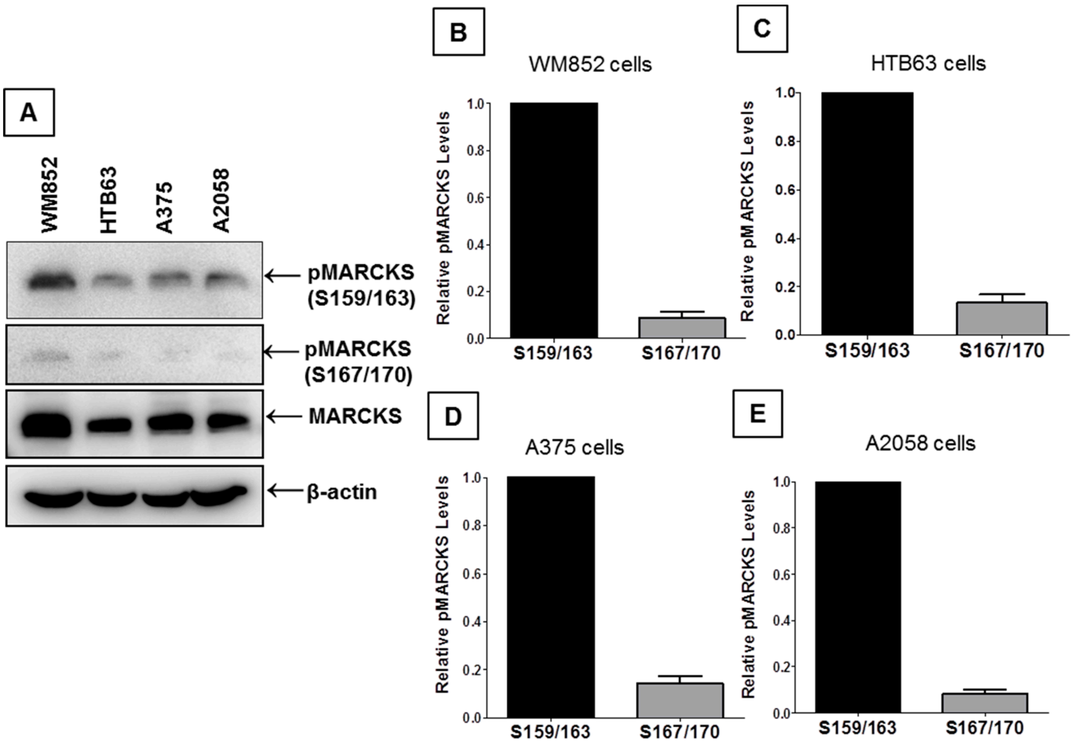

Figure S3: Phosphorylation levels of MARCKS at Ser-159 and Ser-163 are predominant in metastatic melanoma cells. Western blotting was performed as described in the main materials and methods section to evaluate the degree of MARCKS phosphorylation at various 'Serine' residues in different melanoma cell lines. **(A-E)** Representative western blots showing the levels of MARCKS phosphorylation at Ser-159/163 and Ser-167/170 in different melanoma cells. β-Actin was used as a loading control. The graphs (n=4) represent the comparative analyses between the densitometry levels of pMARCKS Ser-159/163 and pMARCKS Ser-167/170 in **(B)** WM852, **(C)** HTB63, **(D)** A375 and **(E)** A2058 melanoma cells.

Supplementary Figure S4

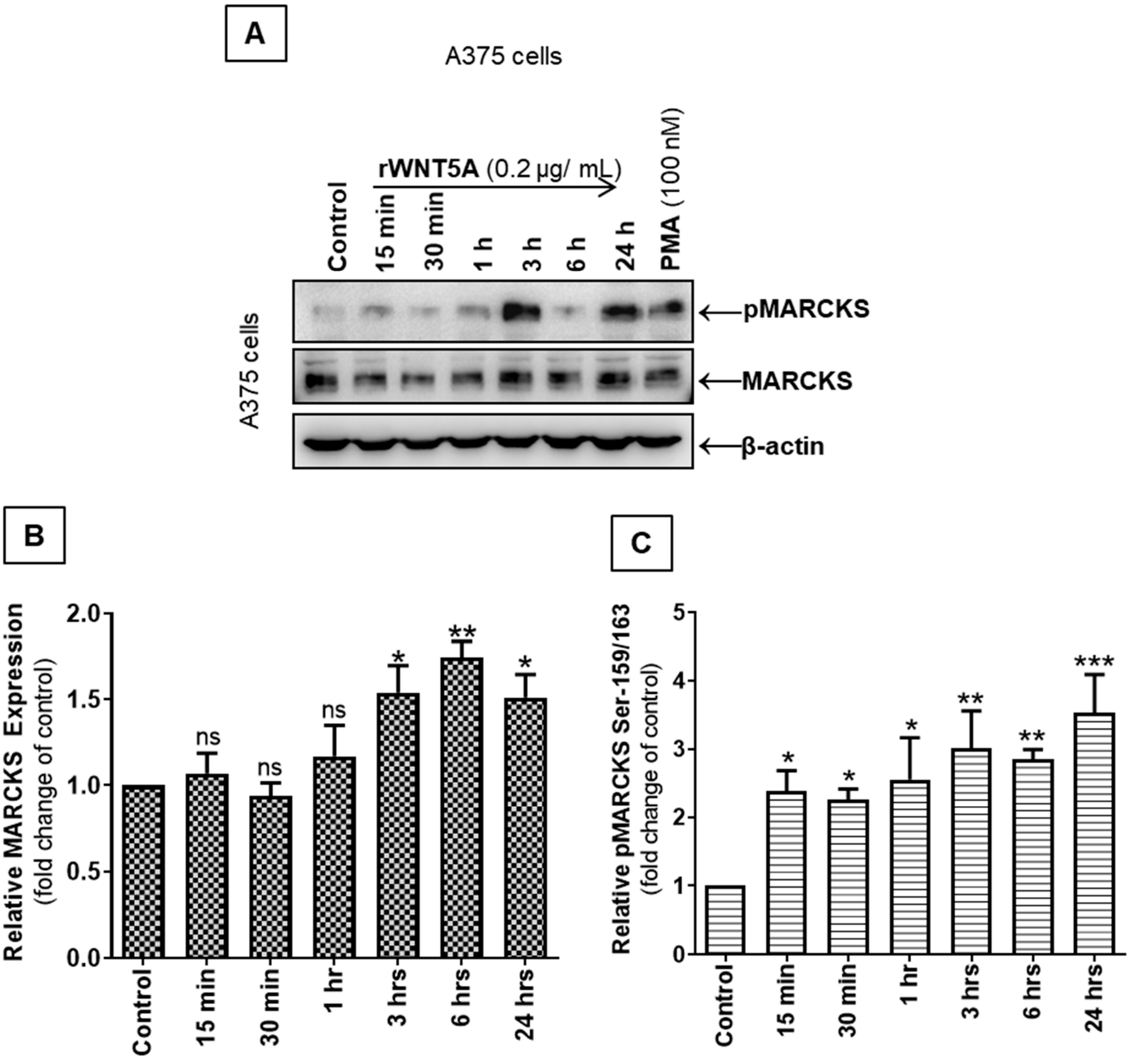

Figure S4: WNT5A signaling increases the expression and phosphorylation of MARCKS in A375 melanoma cells. **(A)** Western blot analysis was performed to evaluate the expression and phosphorylation levels of MARCKS in A375 melanoma cells after stimulation with 0.2 µg/mL rWNT5A protein for the indicated time periods. β-Actin was used as a loading control, and representative blots from four separate experiments are shown here. **(B, C)** The graphs represent the densitometry analyses of the **(B)** expression of MARCKS and **(C)** pMARCKS-Ser-159/163 levels in rWNT5A-treated A375 melanoma cells. The results (n=4) are presented as the means ± S.E.M.; *, p<0.05, **, p<0.001, ***p<0.001.

Supplementary Figure S5

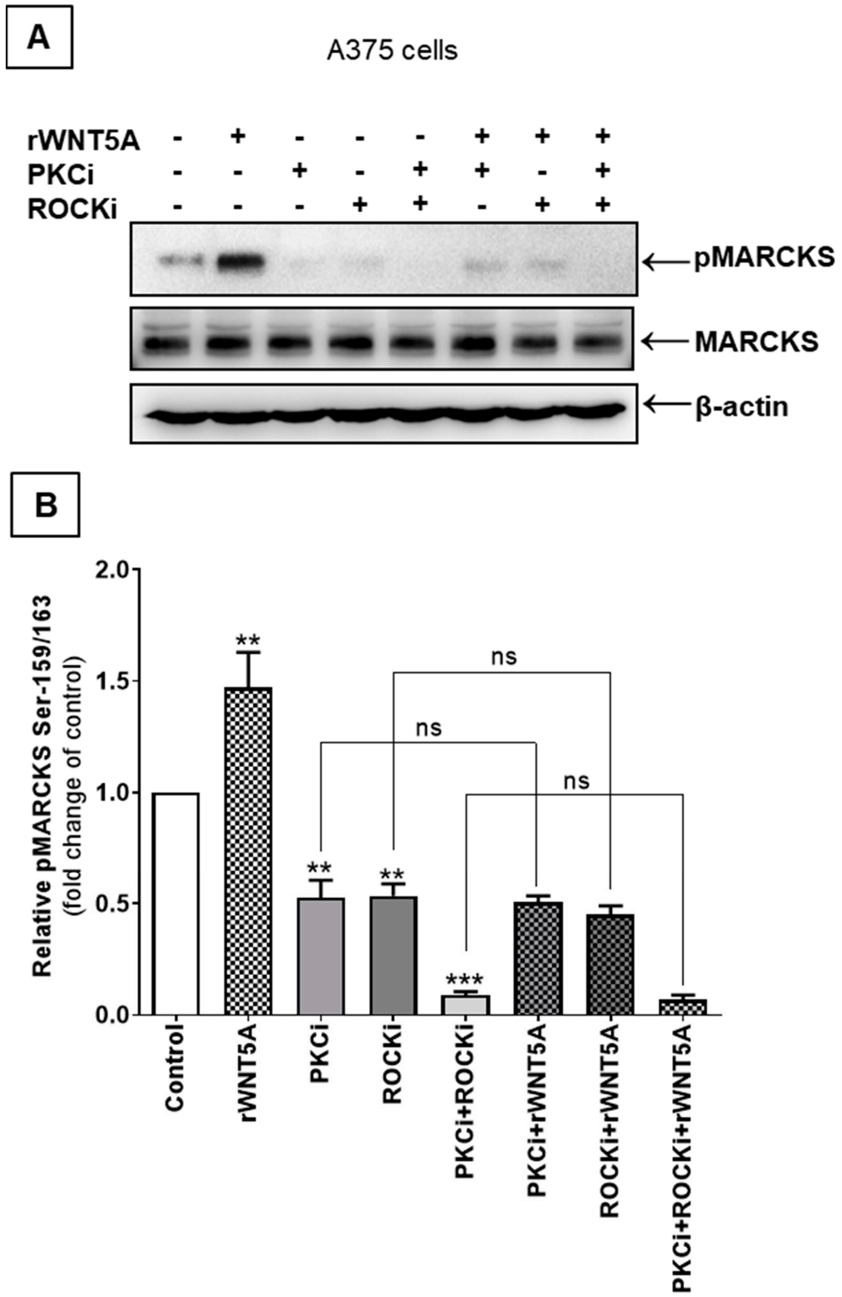

Figure S5: PKC and RhoA-ROCK signaling are also important for WNT5A-mediated phosphorylation of MARCKS in A375 melanoma cells. (A) Western blots showing the phosphorylation levels of MARCKS in PKC and ROCK inhibitor-treated A375 cells with or without rWNT5A stimulation. β-Actin was used as a loading control. **(B)** The graph represents the densitometry analysis of Ser-159/163 phosphorylated MARCKS normalized against total MARCKS using Bio-Rad ImagePro 6.0 software. The results are shown as the mean ± S.E.M.; **, $p < 0.01$, ***, $p < 0.001$.

Whole Blots for Western Blotting Experiments

Figure 1A

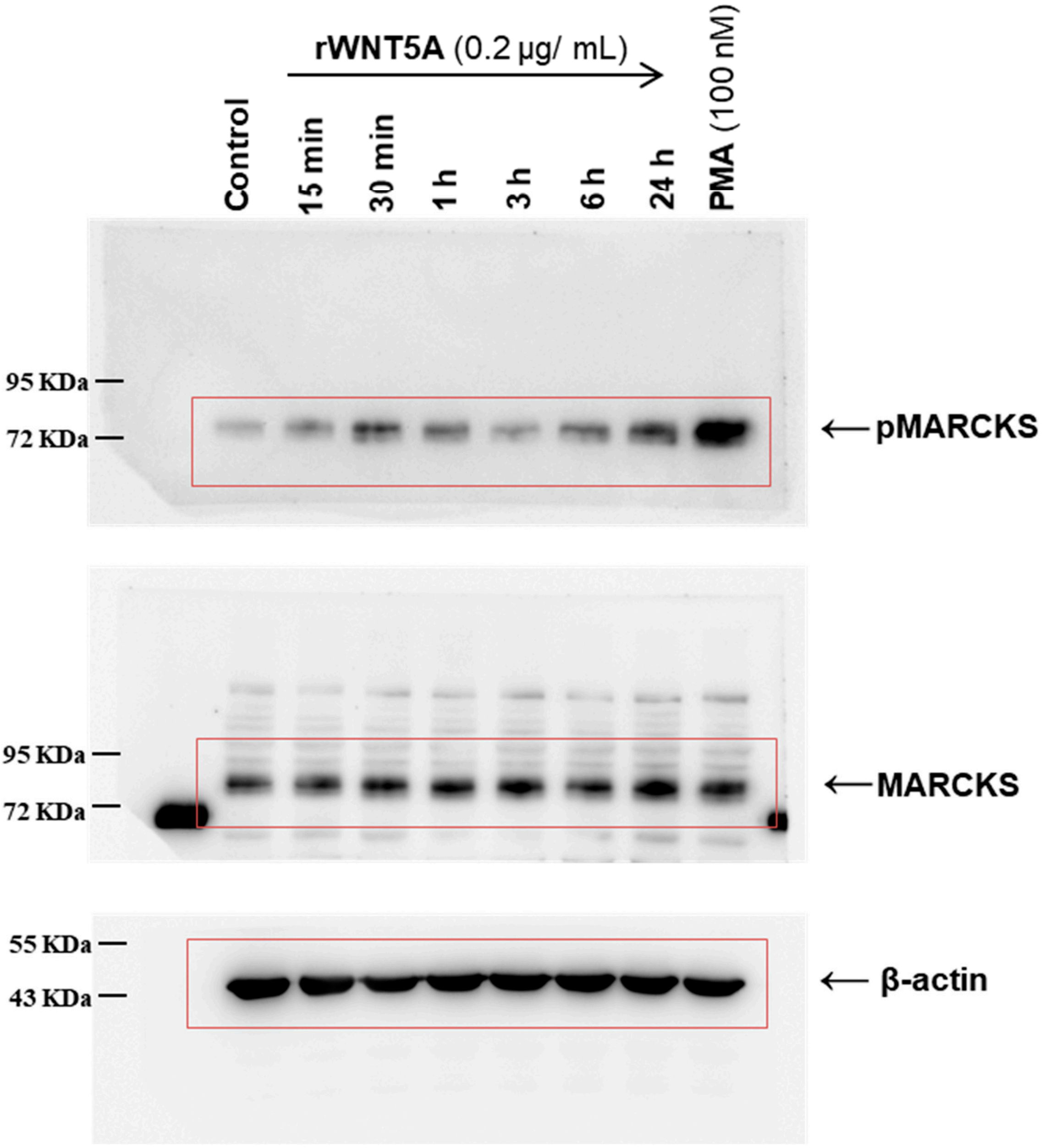

Figure 2A

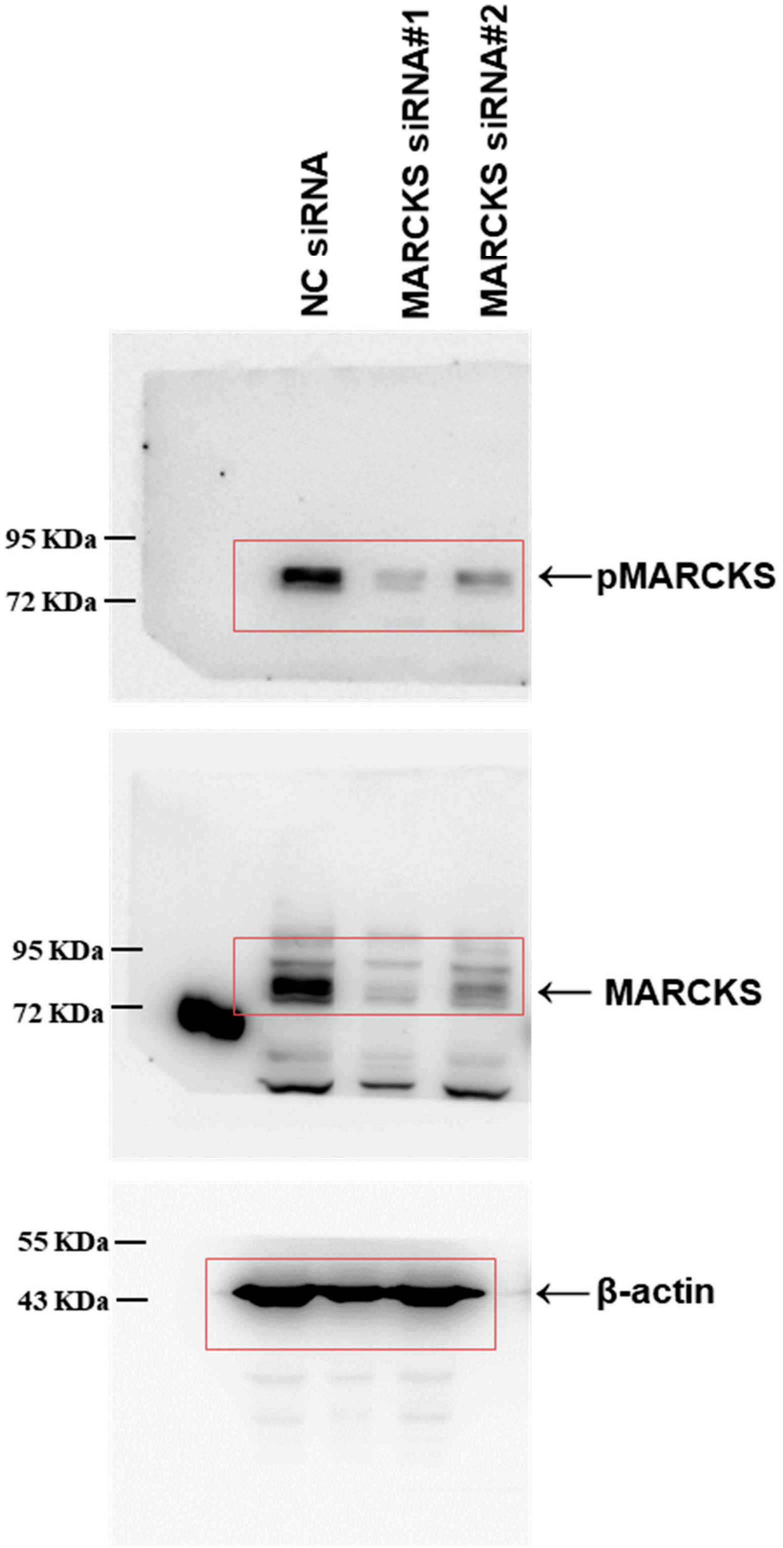
NC siRNA
MARCKS siRNA#1
MARCKS siRNA#2
95 KDa
72 KDa
pMARCKS
95 KDa
72 KDa
MARCKS
55 KDa
43 KDa
β-actin

Figure 3A

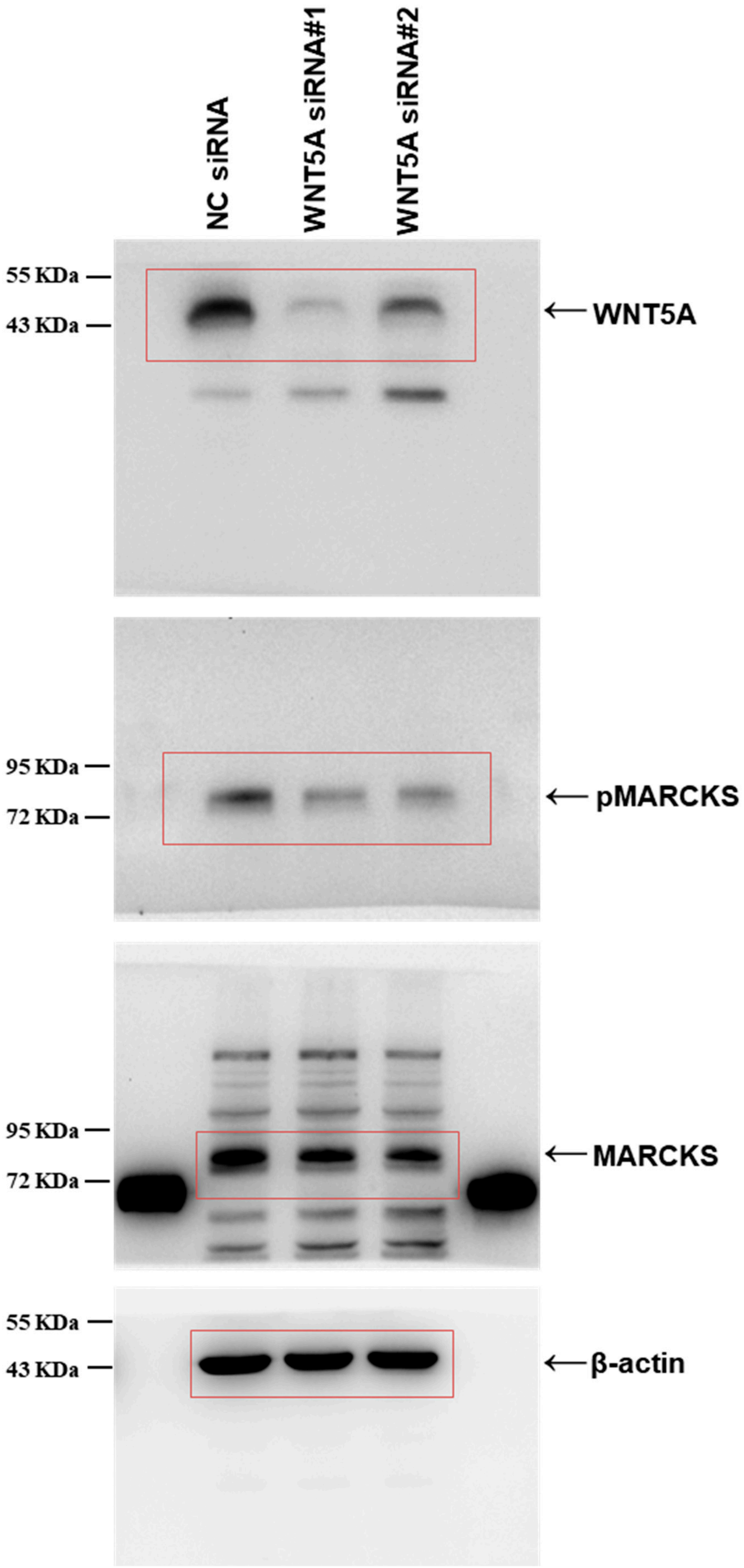
NC siRNA
WNT5A siRNA#1
WNT5A siRNA#2
55 KDa
43 KDa
WNT5A
95 KDa
72 KDa
pMARCKS
95 KDa
72 KDa
MARCKS
55 KDa
43 KDa
β-actin

Figure 4A

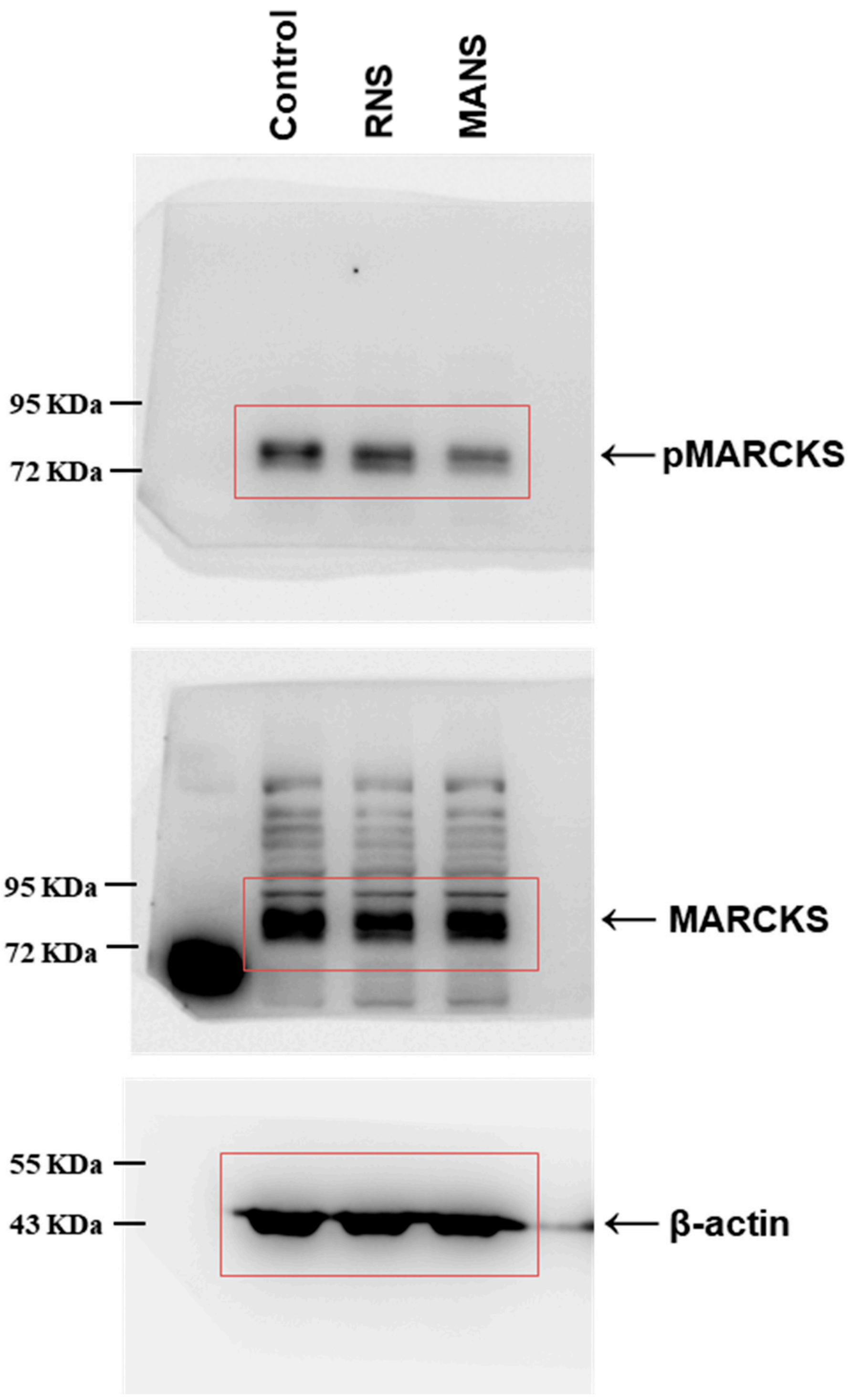

Control
RNS
MANS
95 KDa
72 KDa
pMARCKS
95 KDa
72 KDa
MARCKS
55 KDa
43 KDa
β-actin

Figure 6A

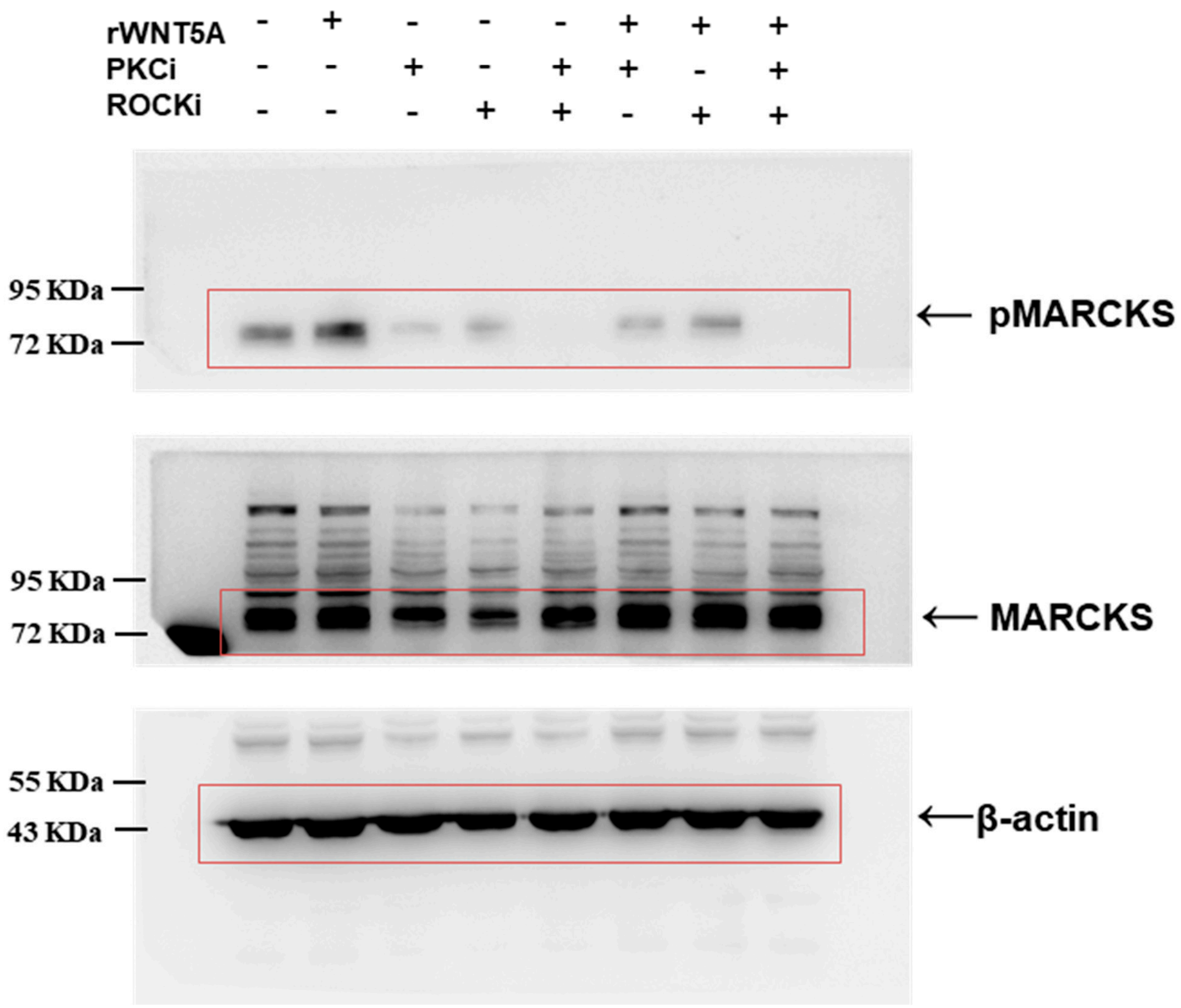
rWNT5A - + - - - + + +
PKCi - - + - + + - +
ROCKi - - - + + - + +
95 KDa
72 KDa
pMARCKS
95 KDa
72 KDa
MARCKS
55 KDa
43 KDa
β-actin

Supplementary Figure S2B

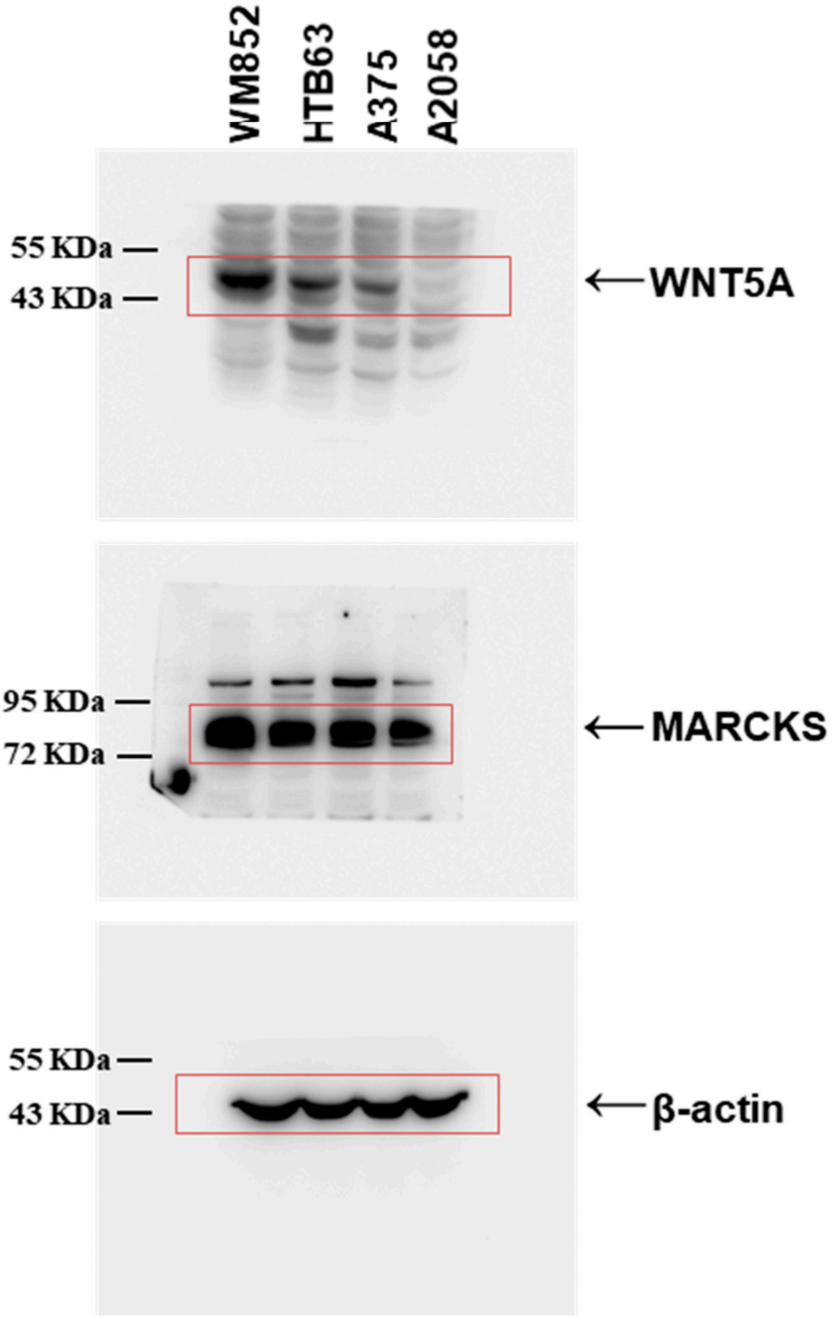

Supplementary Figure S3A

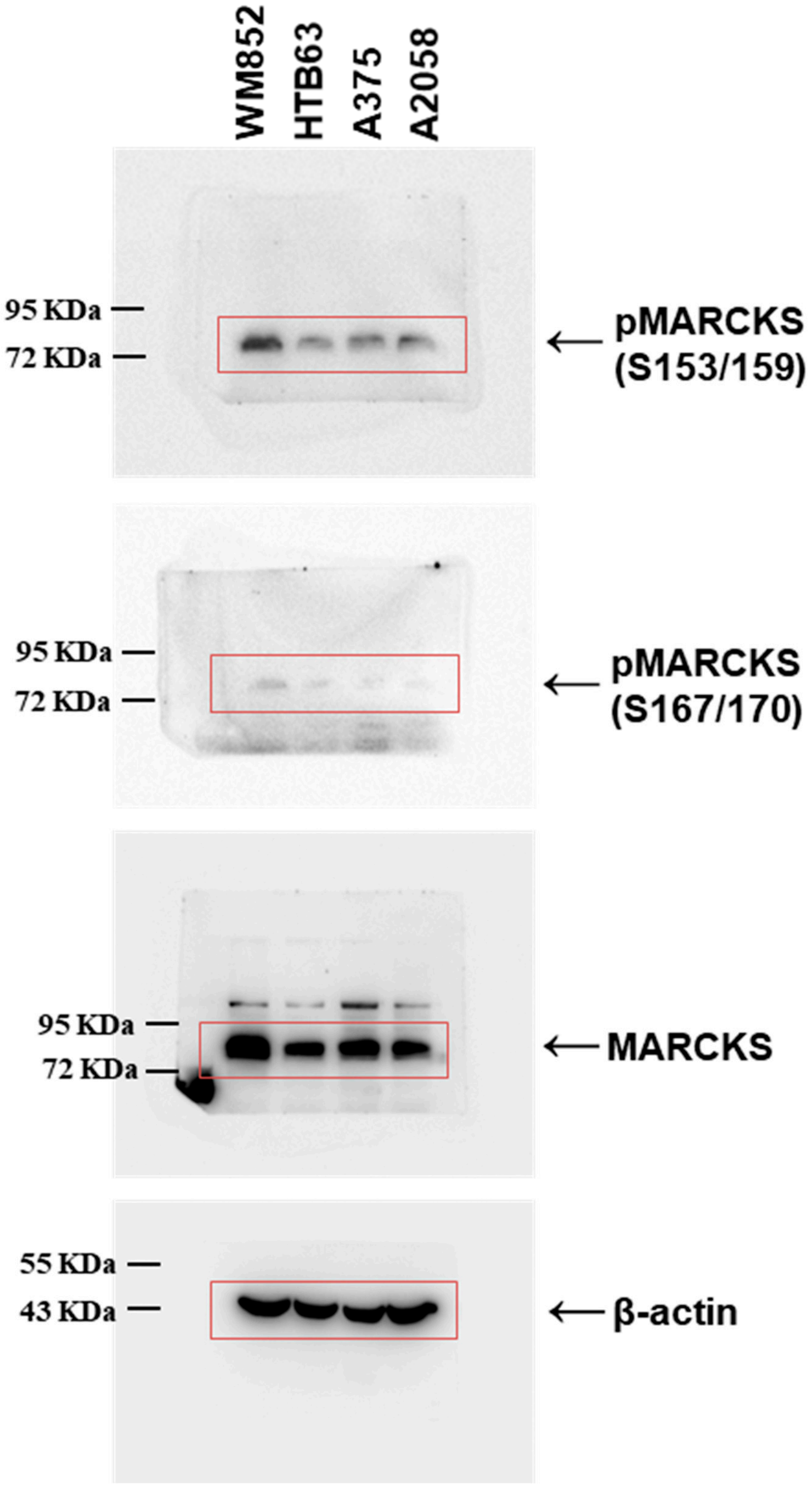

Supplementary Figure S4A

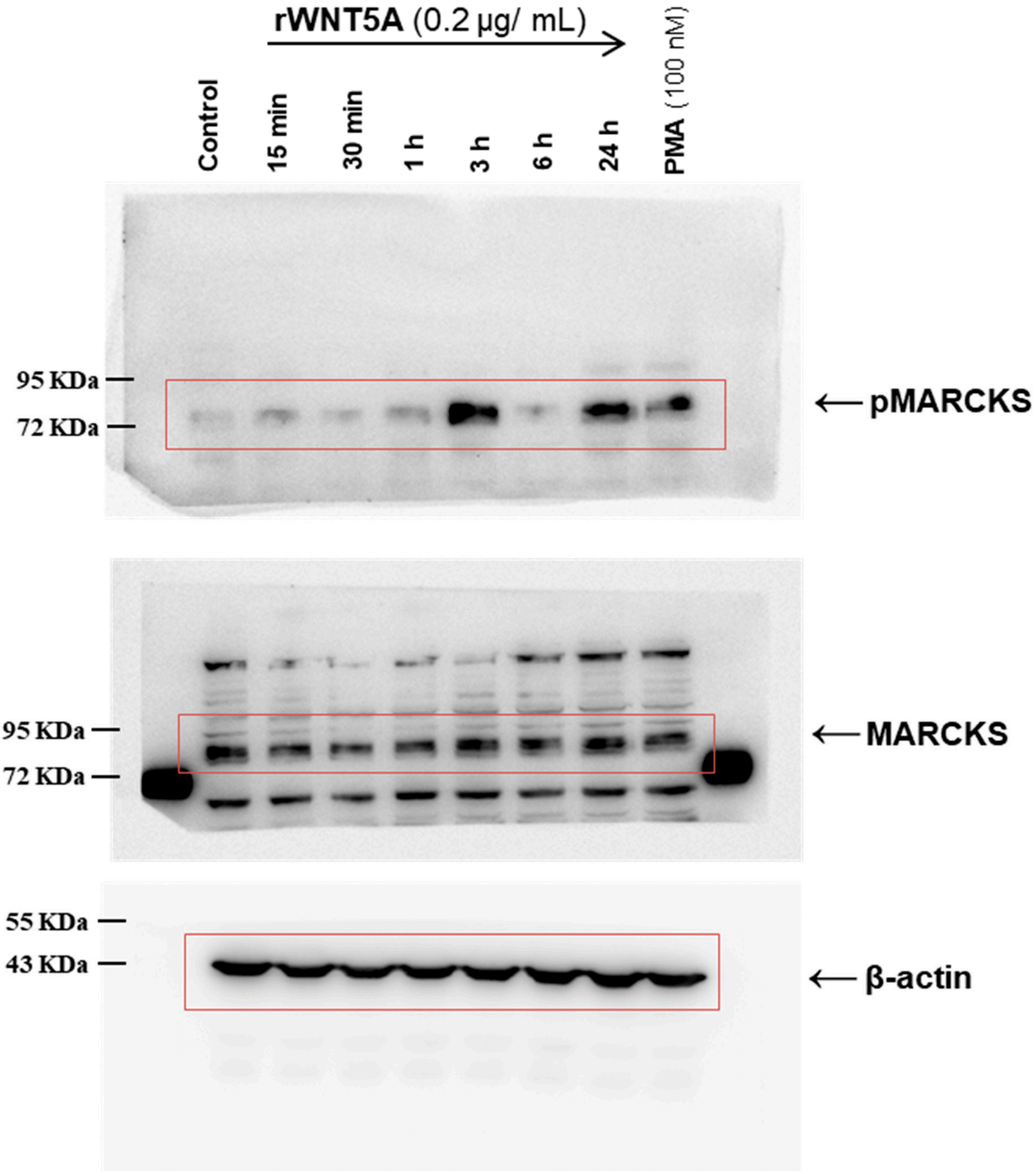

Supplementary Figure S5A

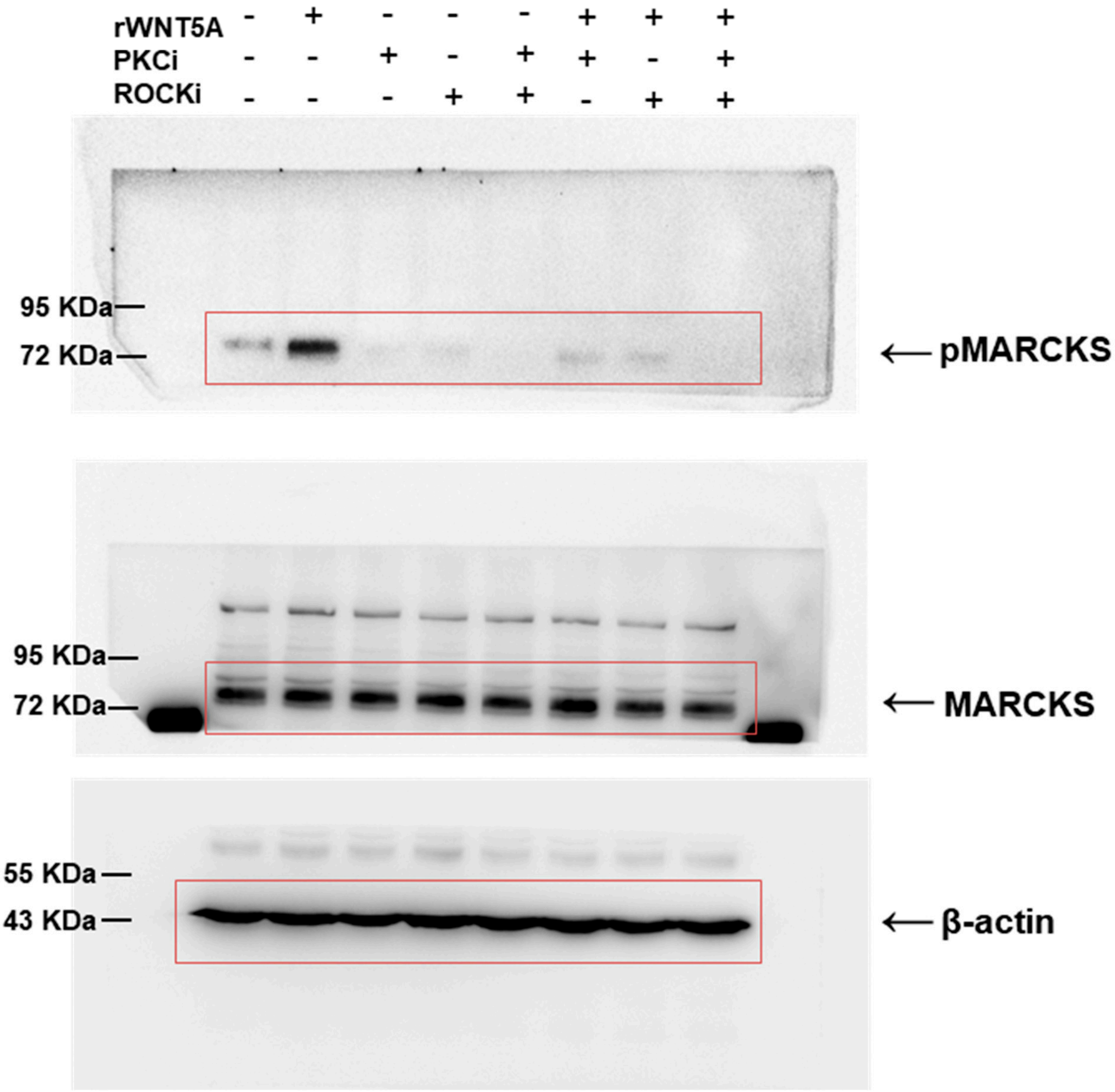

Supplement: Supplementary file 1 [file cancers-12-00346-s001.pdf]
